# Relating Seasonal Hunger and Prevention and Coping Strategies: A Panel Analysis of Malawian Farm Households

**DOI:** 10.1080/00220388.2017.1371296

**Published:** 2017-09-18

**Authors:** C. Leigh Anderson, Travis Reynolds, Joshua D. Merfeld, Pierre Biscaye

**Affiliations:** * Evans School of Public Policy and Governance, University of Washington, Seattle, WA, USA; ** Environmental Studies Program, Colby College, Waterville, ME, USA

## Abstract

Relative to chronic hunger, seasonal hunger in rural and urban areas of Africa is poorly understood. This paper examines the extent and potential correlates of seasonal hunger in Malawi using panel data from 2011–2013. We find that both urban and rural households report seasonal hunger in the pre-harvest months. Certain strategies to smooth consumption, including crop storage and livestock ownership, are associated with fewer months of hunger. In addition, we find that Malawian households that experience seasonal hunger harvest their crops earlier than average – a short-term coping mechanism that can reduce the crop’s yield and nutritional value, possibly perpetuating hunger.

## Introduction

Overcoming chronic hunger and food insecurity has long been acknowledged as essential for reducing global poverty. Both the Millennium Development Goals (MDGs) and the more recent Sustainable Development Goals (SDGs) target hunger (UN, 2016). And yet the United Nations Food and Agriculture Organization (FAO) and the World Food Programme (WFP), which track global and regional trends in hunger and undernourishment, report that nearly 800 million people worldwide remain chronically undernourished (FAO, 2015; WFP, 2015). Though hunger rates are falling globally, progress in sub-Saharan Africa (SSA) has been slower than average. More than one quarter of the world’s hungry now live in SSA as compared to 17 per cent in 1990, and though the percentage affected by hunger is estimated to have fallen from 33 per cent to 23 per cent over the same time period, the total number of hungry has grown with the population (FAO, 2015).

Unlike chronic hunger, which refers to a longer-term food shortage or lack of access to food and may include hunger in the lean season, seasonal hunger is periodic and driven by seasonal variation in climatic conditions (Ayalew, 1997; Gebrehiwot & Van der Veen, 2014; Vaitla, Devereux, & Swan, 2009). Throughout SSA the rural poor depend heavily on subsistence agriculture and, as such, are particularly susceptible to seasonal hunger in the months leading up to the annual harvest (Abdalla, Leonhäuser, Bauer, & Elamin, 2013; Barrett, 1996; Becquey et al., 2012). The effects of seasonality may also spill over to urban populations as food prices rise in response to increasing food scarcity (Chambers, Longhurst, & Pacey, 1981; Garrett & Ruel, 1999).

Several studies discuss seasonal hunger causes and mitigation strategies in selected geographies (Devereux, Sabates-Wheeler, & Longhurst, 2013; Devereux, Vaitla, & Swan, 2008; Hadley, Mulder, & Fitzherbert, 2007; Khandker, Khalily, & Samad, 2012; Mazid & Johnson, 2010; Swan, Hadley, & Cichon, 2010). Despite chronicles of seasonal hunger in SSA dating back to the 1700s (Park, 1799; as cited in Rijpma, 1996) and coverage of periodic seasonal hunger crises from climate or price shocks, however, no estimates of seasonal hunger are systematically compiled at the international level and its current prevalence in the region remains poorly understood.

In Malawi, where over 80 per cent of the population are smallholder farmers, the World Food Programme (WFP) estimates that 6.7 million people (around 37% of the population) experienced hunger between July 2016 and the next harvest in March 2017 following reduced national harvests due to severe drought (WFP, 2017). In 2015, 2.8 million people required food assistance as a result of reduced harvests from a combination of floods and drought (O’Grady, 2016). It is not clear, however, whether seasonal hunger in Malawi is primarily driven by local climatic factors, or if other household and farm characteristics are also factors.

This paper contributes to the body of evidence on the extent and correlates of seasonal hunger in SSA using panel data from the Malawi Integrated Household Panel Survey (IHPS). A large panel sample of rural and urban households across two survey waves (2010–2011, 2013) allows us to: (1) examine the extent of hunger in general and seasonal hunger in particular for rural and urban households over time, (2) evaluate whether seasonal hunger is associated with local climatic factors, household and farm character istics, and strategies to smooth consumption and diversify income, and (3) investigate whether seasonal hunger might induce agricultural households to harvest early as a coping strategy.

Our findings suggest that both urban and rural households in Malawi experience hunger in the pre-harvest months, with seasonal hunger rates as high as 57 per cent for rural households and 36 per cent for urban households. We also find evidence that some household strategies to smooth consumption, including crop storage and livestock ownership, are associated with fewer months of seasonal hunger. Finally, we find that Malawian households experiencing seasonal hunger appear to frequently adopt early harvesting as a coping mechanism – a practice with potentially detrimental short- and long-term consequences for crop yields, household incomes, and nutrition.

The rest of the paper is organised as follows. We first review the relevant literature on the causes and consequences of seasonal hunger and on potential prevention and coping strategies. We then discuss the data and methods before presenting results on the extent of seasonal and chronic hunger in Malawi and from fixed effects regressions exploring the correlates of seasonal hunger and testing its hypothesised relationship to early harvesting. The final section summarises the findings and policy implications.

## Background

Seasonal hunger occurs when an individual has limited access to food during the months prior to the annual harvest (Ayalew, 1997; Devereux, 2009; Gebrehiwot & Van der Veen, 2014).^1^ In subsistence farm households in SSA both food and income from crop sales are typically most plentiful immediately after the harvest. In contrast, particularly in the months immediately preceding harvest – sometimes called the ‘hunger season’ or ‘lean season’ – subsistence households may have run down their stores of grain and savings while energy demands remain high, leading to hunger and compromising productivity (Behrman & Deolalikar, 1989; Strauss, 1986). Seasonal hunger can be exacerbated by unusual variations in weather patterns or by household shocks such as household member unemployment or sickness (Barrett, 2010; Bogale, 2012; Gebrehiwot & Van der Veen, 2014; M’Kaibi, Steyn, Ochola, & Du Plessis, 2015).

Given the agricultural basis of seasonal hunger, it is often assumed to impact only rural farmers who directly depend on agriculture for their food and livelihoods. But people living in urban areas can also experience seasonal hunger if availability declines and prices rise for purchased food in the months preceding the harvest (Garrett & Ruel, 1999). Because of multiple food sources and the consequent greater market access and food variety, however, urban seasonal hunger is less common than rural seasonal hunger (Barrett, 1996; Becquey et al., 2012).

In Malawi, chronic hunger for much of the population in recent years is attributed to challenges with inadequate infrastructure, infertile soils, ineffectual government policies, and changes in weather patterns (Prior, 2013). More frequent and intense climate shocks, such as episodes of drought or severe flooding, have contributed to recurring seasonal hunger and famine (Asfaw & Maggio, 2017; O’Grady, 2016; WFP, 2017). Although there are estimates of the number of hungry individuals and evidence of seasonal hunger in Malawi, it is not clear what proportion of the population experiences chronic as opposed to seasonal hunger, and whether there are differences in hunger between rural and urban areas.

We contribute to the literature on seasonal hunger by using nationally-representative survey data from 2010–2011 and 2013 to examine the extent of chronic and seasonal hunger in Malawi among urban and rural populations. We then evaluate whether certain household and farm characteristics and strategies are associated with seasonal hunger in Malawi, or if hunger in the months leading up to harvest is primarily correlated with local climatic factors. In particular, we consider the effects of various strategies to reduce the risk of seasonal hunger, including crop diversification, growing off-season or permanent crops, improved farm management practices, livestock ownership, crop storage, crop sales, and working for wages.

Vulnerability to seasonal hunger may lead farm households to take steps to smooth consumption between harvests. One of the more common strategies is farm diversification, which includes planting ‘off-season’ or perennial crops as well as carrying livestock (Maxwell, 1996; Mayanja, Rubaire-Akiiki, Greiner, & Morton, 2015; Megersa, Markemann, Angassa, & Zárate, 2014; Morris, Mendez, & Olson, 2013; Rademacher-Schulz, Schraven, & Mahama, 2014; Rosenzweig & Wolpin, 1993). Higher crop diversity on a smallholder’s land has been associated with reduced levels of seasonal hunger and malnutrition (Abdalla et al., 2013; Afifi et al., 2015; Bacon et al., 2014; Devereux, 2009). Certain crops, like sweet potatoes and garden vegetables, make off-season harvesting of new food sources possible (Arimond et al., 2011; Krishnal & Weerahewa, 2014), whereas early maturing seed varieties help to reduce the wait before harvests (Herforth, 2010; Keding & Cogill, 2013; Mazid & Johnson, 2010; Mburu, Kung’u, & Muriuki, 2015; Powell et al., 2015; Zug, 2006).

In addition to smoothing consumption directly, planting multiple crops helps to spread the risk of lost income for households that sell crops (Reardon, 1997). In some cases, farm households specialising in cash crops (resulting in less diverse crop portfolios but increased ability to participate in markets) have been found to be more vulnerable to seasonal variations in hunger (Fleuret & Fleuret, 1980). One recent study finds that increased incomes from cash cropping may not be sufficient for improved nutritional outcomes in Malawi due to differences in households’ ability to sell harvest and/or buy marketed goods (Radchenko & Corral, 2017).

Improved agricultural management, including irrigation techniques and fertiliser usage as well as conservation agricultural methods (Afifi et al., 2015; Nyanga, 2012), is another strategy for preventing hunger, based on the premise that increasing crop yields can increase the likelihood that food is available in the months pre-harvest (Rademacher-Schulz et al., 2014). The Malawi Farm Input Subsidy Program (FISP) has contributed to increased maize yields by providing coupon-vouchers for fertiliser and improved seed (Arndt, Pauw, & Thurlow, 2016), although the programme has been criticised for not reaching targeted poor and vulnerable households (Chibwana, Fisher, Jumbe, Masters, & Shively, 2010). Resilient and improved crop varieties can also increase average yields from crops, making a greater store of food available to stretch into the lean season (Edeh & Gyimah-Brempong, 2015).

When markets to purchase food are available, households can smooth consumption through other channels, such as off-farm or non-farm employment. Off-farm income opportunities help to fill seasonal income gaps by separating households from some of the risks associated with agriculture (Afifi et al., 2015; Daie & Woldtsadik, 2015; Rademacher-Schulz et al., 2014; Sibhatu, Krishna, & Qaim, 2015). Alternatively, the household may trade labour for money or food, or household members might temporarily migrate to another area in search of work (Afifi et al., 2015; Hadley & Patil, 2008; Maxwell, 1996; Mayanja et al., 2015; Morris et al., 2013; Rademacher-Schulz et al., 2014; Zug, 2006). Similar to the effects for urban food consumers, however, rural purchasers of food are likely to face higher prices in the lean season (Barrett, 1996). Income diversification is common in Malawi, with many rural households relying on non-farm income in addition to cash crops (Masanjala, 2006), and these alternative livelihood strategies have helped some households to avoid and cope with seasonal food deficits (Orr, Mwale, & Saiti-Chitsonga, 2009).

Finally, in addition to the above ex-ante strategies to mitigate or avoid seasonal hunger, several ex-post responses to seasonal hunger have been reported. Households may eat less preferable or lower quality foods during the hungry season or rely on collected wild foods to fill caloric gaps (D’Souza & Jolliffe, 2012; Daie & Woldtsadik, 2015; Edeh & Gyimah-Brempong, 2015; Hadley & Patil, 2008; Maxwell, 1996; Mayanja et al., 2015). In extreme cases, household members may skip meals or eat smaller portion sizes, or reduce household size through early marriage or sending members to live with relatives or friends (Edeh & Gyimah-Brempong, 2015; Hadley & Patil, 2008; Khandker et al., 2012; Maxwell, 1996; Mayanja et al., 2015; Rademacher-Schulz et al., 2014). Households have been observed to sell assets – including livestock – in order to purchase food (Heltberg,  Hossain, Reva, & Turk, 2013; Mayanja et al., 2015; Rademacher-Schulz et al., 2014; Rosenzweig & Wolpin, 1993; Zug, 2006). Additionally, seasonally hungry households may borrow food or money to purchase food from either relatives or friends (Edeh & Gyimah-Brempong, 2015; Hadley & Patil, 2008; Maxwell, 1996; Mayanja et al., 2015; Morris et al., 2013; Zug, 2006).

Households in Malawi have been observed to employ many of these strategies to smooth consumption. In some areas of Malawi, households rely on extracting forest goods to help smooth consumption following negative income shocks (Fisher & Shively, 2005). Additionally, *ganyu* labour, where a person labours for family members or friends at extremely low wages, is also common (Harrigan, 2008; Orr et al., 2009). Finally, skipping meals and selling household assets are commonly observed during the hungry season in Malawi (Ellis & Manda, 2012; Harrigan, 2008). Government poverty-targeted social cash transfers also support poor farmers to cope with seasonal hunger (Ellis & Maliro, 2013).

Another hypothesised but not empirically established short-run on-farm response of seasonal hunger is early harvesting of crops before they mature (Wagstaff & Lindelow, 2014). While little literature has explicitly studied the effects of early harvest in developing countries, there is evidence that harvesting immature crops reduces the crop’s potential yield and nutritional value (Cahill et al., 2014; Kamruzzaman & Islam, 2008; Lindsay et al., 2016). As a result, households that harvest their crops early might increase their vulnerability to ongoing seasonal hunger. Our final contribution to the literature on seasonal hunger is thus to test the association between seasonal hunger and early harvesting in Malawi, to determine whether there is evidence of this hypothesised short-term coping strategy with potential long-term consequences.

## Data

We use data from the Malawi Integrated Household Panel Survey (IHPS), a part of the World Bank’s Living Standards Measurement Study-Integrated Surveys on Agriculture (LSMS-ISA). Two waves of Malawi IHPS data have been collected by the Malawi National Statistics Office (NSO). The first wave (interviewed March 2010–March 2011) included a large cross-section of more than 9000 households, of which we include only the 3227 panel households that were tracked and interviewed again for the second wave, between April and October of 2013. Tracking was conducted at the individual level, so the Wave 2 sample size totals 3980 households when new households that split from Wave 1 panel households are included. The survey used a two-stage stratified random sample, with enumeration areas (EAs) based on the 2008 Malawi Census as the primary sampling unit. Sixteen households per EA were randomly selected and interviewed. The panel of respondents included in both survey waves is representative at the national, urban/rural, and regional levels (NSO, 2014).

The survey includes a wide variety of questions on household demographics, farm activities, and livelihoods. Household and farm-level summary statistics for both survey waves are shown in Table 1.

**Table 1.** Summary statistics

	Wave 1		Wave 2	
	mean	sd	mean	sd
Age of household head	42.040	(16.012)	42.4 71	(15.777)
Education of household head (years)	7.680	(3.942)	7.824	(4.072)
Male household head	0.780	(0.414)	0.771	(0.421)
Household size	4.798	(2.325)	5.063	(2.362)
HH distance to nearest road (km)	7.559	(8.950)	7.658	(9.289)
July-June total rainfall (mm), current season	787.127	(121.596)	822.693	(89.363)
Acres owned	2.206	(9.823)	3.665	(36.878)
Crop count	1.742	(1.336)	1.941	(1.598)
Used any organic fertiliser	0.172		0.201	
Used any inorganic fertiliser	0.801		0.748	
Simpson index in previous season (planted acres)	0.337	(0.299)	0.355	(0.295)
Grew off-season or permanent Crop	0.352		0.360	
Poultry (count)	3.101	(6.723)	3.065	(14.695)
Other livestock (count)	1.424	(4.179)	1.296	(3.933)
Stored annual or permanent Crop	0.665		0.610	
Remittances and gifts (log of total cash received – MK)	3.051	(4.040)	4.284	(4.602)
Household member worked for wage	0.612		0.625	
Sold annual or permanent crop	0.076		0.112	
Rural household	0.736		0.738	
Agricultural household	0.822		0.789	
Observations	3227		3980	

Farm and household characteristics are similar across the two survey waves, reflecting the panel nature of the survey. Households in Malawi average just under five household members in the first wave growing to slightly more than five in the second. These households also tend to live in rural areas (73%), with an average distance to the nearest road of more than seven kilometres. The average household grows slightly fewer than two different crops on just over two acres of land in Wave 1, increasing to more than 3.6 acres in Wave 2.^2^ Inorganic fertiliser use is very common, not surprising as Malawi has one of the largest input subsidy programmes in SSA (the FISP). Many households (35–36%) grow a permanent or off-season crop. Ownership of mammal (including cows, pigs, sheep, and goats) and poultry (primarily chickens) livestock is relatively common, with households owning just over three poultry livestock and under 1.5 other livestock on average. In addition, over 60 per cent of households include household members that perform wage work. Over 60 per cent of households store at least part of their harvest, but overall households are unlikely to sell crops; less than 8 per cent of households reported selling an annual or permanent crop in Wave 1, and 11 per cent in Wave 2. On average respondent households experienced more annual rainfall and reported receiving more remittances in Wave 2.

## Methods

We first use respondent reports of the months in which they were hungry to analyse the extent and timing of hunger in rural and urban households in the two survey waves. We then examine the prevalence and length of seasonal as opposed to chronic hunger for urban and rural households using a common ‘seasonal hunger’ period based on the most common month of first harvest in the sample.

We measure seasonal hunger with a value between zero and four based on the number of months when a household reports experiencing hunger during the three months prior to harvest, plus the month of first harvest by that household. This definition of seasonal hunger attempts to capture hunger gradations through length of deprivation, but it does not measure depth. We use a simple count rather than consecutive months of hunger pre-harvest as the ‘hunger’ months may be interrupted (for example, if a household experiences hunger in January, sells an asset or borrows money in February to smooth consumption, then is hungry again by March prior to April’s harvest). A limitation of the data is that households are asked to report hunger and date of first crop harvest by month, rather than daily or weekly, potentially masking considerable intra-month variation and resulting in responses tied to the beginning of a month being treated equivalently to responses up to 30 days later. However, robustness checks suggest model results are not sensitive to alternate definitions of seasonal hunger.^3^ Moreover, we consider this definition to be conservative in that it will more likely underestimate than overestimate the extent of hunger due to seasonal causes, since very food-insecure households may experience hunger for a season of longer than four months if they run out of crop and income stores relatively shortly following the previous year’s harvest.

After reporting descriptive statistics for seasonal hunger, we perform several analyses. In the first set of analyses, the dependent variable of interest is the count variable of seasonal hunger. In order to explore associations between seasonal hunger and hypothesised drivers including household and farm characteristics, climate variations, and on- and off-farm prevention strategies, we estimate ordered logit regressions of the form:

(1) $$y_{h}=\alpha +\beta X_{h}+\varepsilon_{h}$$

where $y_{h}$ is months of seasonal hunger and $X_{h}$ is a vector of covariates. We estimate these models for the cross-section of agricultural households^4^ in each wave as well as for a pooled sample with enumeration area (EA)- and wave-level fixed effects in order to control for time-invariant EA-level characteristics such as soil quality, community storage facilities, extension, and other localised policies or factors potentially associated with seasonal hunger.

We estimate two models for each of these regressions, the first including only household- and farm-level control variables, and the second including strategies commonly reported in the literature for smoothing consumption and reducing vulnerability to seasonal hunger (arguably endogenously).^5^ The household- and farm-level control variables include the age, education, and gender of the household head, the size of the household, the distance of the household to the nearest road, and the number of acres owned. Given the presence of the FISP in Malawi and the relationship between fertiliser use and yield, we also include whether the household used any organic or inorganic fertiliser. The second set of models adds indicators of on- and off-farm strategies – again as theorised in the literature – used by households to mitigate seasonal hunger. Indicators include diversifying crop portfolios (measured by the Simpson index, with a higher value representing greater crop diversity^6^), receiving remittances, counts of poultry and other livestock, and dummy variables taking a value of 1 if the household cultivated off-season or permanent crops such as tree fruits and roots, tubers, or bananas, stored harvested crops for delayed consumption or sale, sold any crops, and had any household member work for a wage.

We do not use household fixed effects in these analyses because we are interested in a number of variables that are time invariant (for example, education of household head, distance to nearest road). Both waves of the Malawi IHPS, however, include two different agricultural modules. The first module (Agricultural Module B) asks about the agricultural season from the previous year.^7^ The second module (Agricultural Module G) asks about the agricultural season from the current year.^8^ For all regressions, we use information on farm decisions from the previous year’s agricultural season, as these decisions would precede the period in which households might experience seasonal hunger leading up to the current agricultural season.

In order to account for the influence of climatic factors on harvest volumes, we also include total rainfall in the reference growing season at the household level as a control variable in all regressions. Malawi’s agriculture is predominately rain-fed, and current food crises have been attributed to changes in rain patterns (O’Grady, 2016). If seasonal hunger variation is due solely to changes in crop production driven by rainfall, the potential for remedial measures is limited. The rainfall variables are provided as a part of the IHPS data, and are constructed from annual WorldClim and National Oceanic and Atmospheric Administration (NOAA) data.

Finally, we explore the effects of seasonal hunger following the previous agricultural season on timing of the first harvest in the current agricultural season to look for evidence of the hypothesised coping strategy of harvesting early. Here we take advantage of the panel nature of the data and use a household fixed effects model to control for time-invariant household characteristics, and include household size, gender of household head, acres planted, counts of poultry and other livestock, and total rainfall in the reference growing season. We estimate regressions in the form:

(2) $$y_{ht}=\alpha +\phi_{h}+\delta T_{ht}+\beta X_{ht}+\varepsilon_{ht}$$

where $y_{ht}$ is the month of first harvest of any crop from any plot in the current agricultural season in household *h* in wave *t*, $\phi_{h}$ is a household dummy, $T_{ht}$ is a count of the number of months the household was hungry in the four months preceding and including the month of first harvest, $X_{ht}$ is a vector of control variables, and $\varepsilon_{ht}$ is a household-specific error term.^9^ We also run the same regression looking at the month of first maize harvest, as maize is one of the most commonly cultivated crops in Malawi. In these regressions, we include only households appearing in both survey waves.

While the outcome variable is the month of harvest, we choose to use OLS to estimate this last set of regressions for one main reason. Since we have only two waves of data, the household fixed effects inject a substantial number of additional parameters into the model relative to the number of observations. As such, non-linear models – like ordered logit – could result in biased coefficient estimates due to the well-documented incidental parameters problem (Greene, 2008; Wooldridge, 2010). Nonetheless, we also present ordered logit results in the Appendix to help allay concerns regarding the use of OLS with an ordinal outcome variable.

## Results

**Figure 1.**
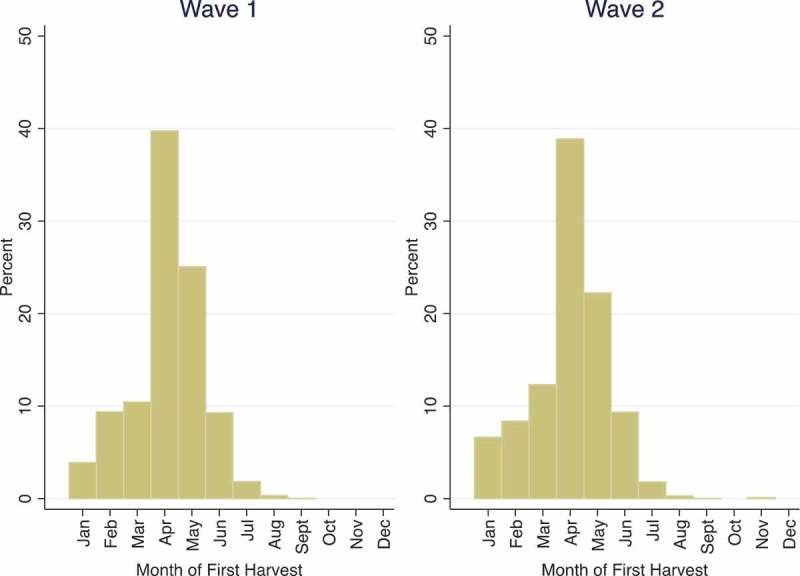
Month of household first harvest.

Around 80 per cent of the households surveyed in Malawi cultivated crops (82% in Wave 1, 79% in Wave 2). The reported date of first harvest allows us to assess when seasonal hunger is likely to be most prevalent for these agricultural households, and also for non-agricultural households for whom the price of purchased food fluctuates in part with harvest timing and output. The rainy season in Malawi begins late in the year and normally ends in the first few months of the next year. Coincident with this rainfall pattern, the data indicate that nearly 40 per cent of households begin to harvest in April, although there is some variation (Figure 1).

**Figure 2.**
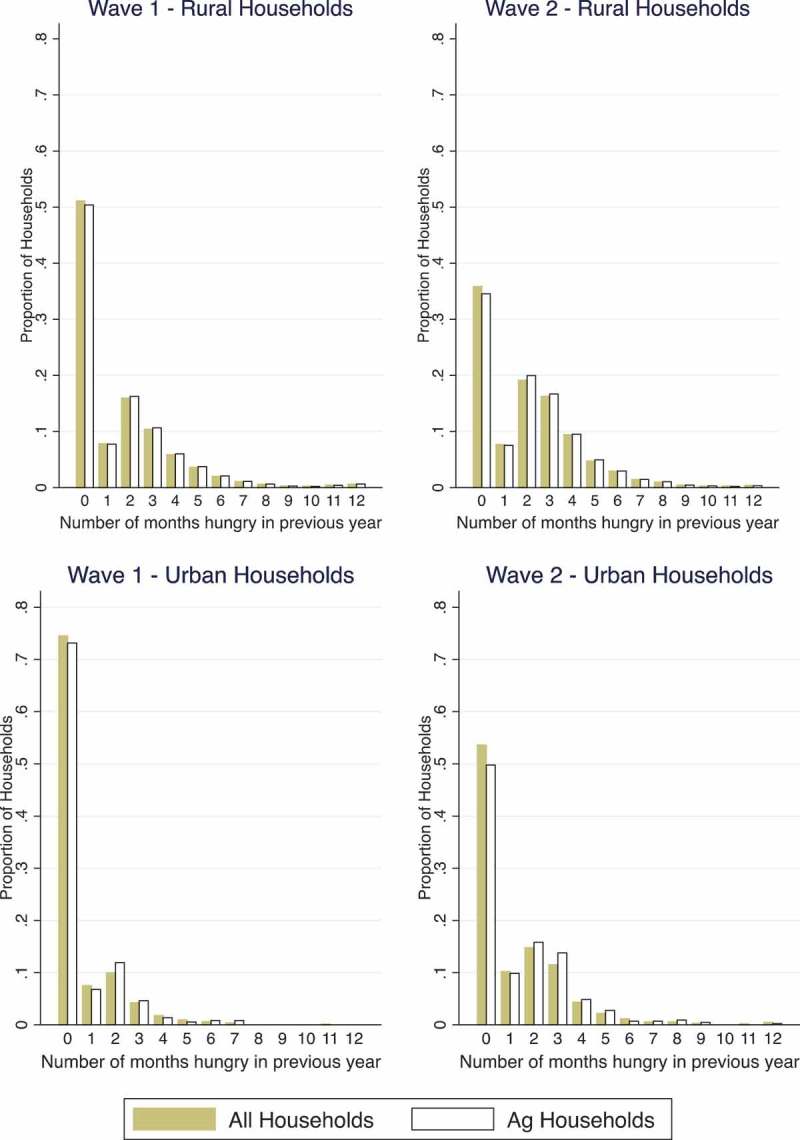
Number of months hungry in rural and urban households.

While Figure 1 only includes agricultural households, 27 per cent of households in the sample are classified as urban. Most urban households are non-agricultural households, while most rural households are agricultural households. As highlighted in Figure 2, hunger is more common in rural households than urban households: more than 70 per cent of urban households during Wave 1 of the survey reported no months of hunger in the year preceding the survey, compared to 50 per cent of rural households. We also see this pattern in Wave 2, though the difference is smaller and both rural and urban households reported a higher number of months of hunger than in the Wave 1. Agricultural households report slightly more months of hunger than non-agricultural households in both urban and rural settings.

**Figure 3.**
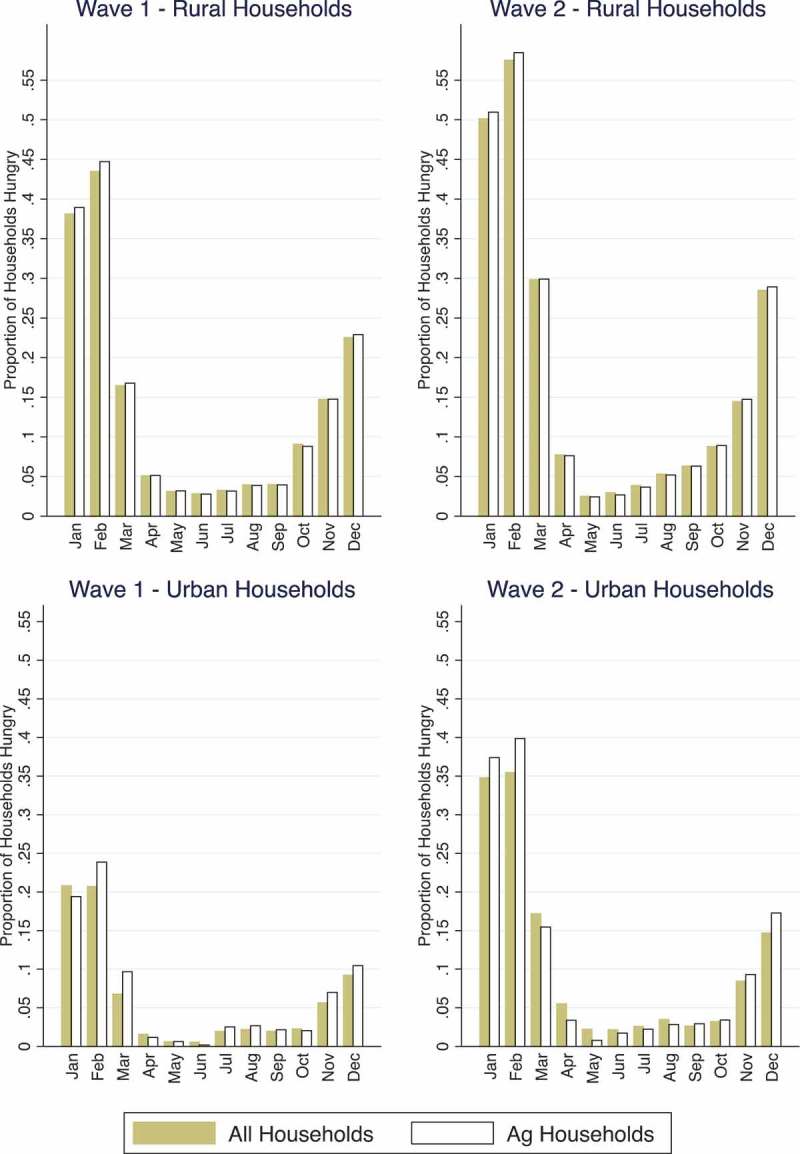
Hunger by month in rural and urban households.

Figure 3 illustrates the prevalence of seasonal hunger in Malawi. The y-axis shows the proportion of households that reported any hunger in the months plotted on the x-axis. Hunger is most prevalent in the months before April (the most common month of first harvest in Malawi), especially January and February. Seasonal hunger patterns are almost identical – although the magnitudes are not – among rural and urban households, with hunger being most common in February and least common in the months immediately following the April harvest.

Table 2 compares seasonal and non-seasonal hunger for rural and urban households. Here we use a common four-month period (January to April) to measure seasonal hunger (rather than considering each household’s own month of first harvest) in order to be able to include non-agricultural households. Panel A presents statistics on seasonal hunger for urban and rural households during this four-month period. Approximately 37 per cent of urban households reported any hunger in January to April, and the average household reported 0.747 months of hunger. Among rural households, over 57 per cent reported any seasonal hunger, and households reported an average of 1.261 months of hunger. Finally, among urban households that reported no months of hunger between May and December, approximately 29 per cent reported at least one month of seasonal hunger (meaning these households experienced seasonal hunger only, as opposed to chronic hunger). Among rural households, the number of non-chronically hungry households that reported seasonal hunger jumps to more than 43 per cent.

**Table 2.** Extent of seasonal and chronic hunger in urban and rural households

	(1)	(2)
	Urban Households	Rural Households
	mean/count	mean/count
Panel A: Seasonal months – January through April		
Hunger in seasonal months (indicator)	0.369	0.571
	1892	5310
Hunger in seasonal months (count)	0.747	1.261
	1892	5310
Hunger in seasonal months if no hunger in non-seasonal months (indicator)	0.292	0.432
	1602	3620
Panel B: Non-seasonal months – May through December		
Hunger in non-seasonal months (indicator)	0.181	0.293
	1982	5310
Hunger in non-seasonal months (count)	0.330	0.685
	1982	5310
Hunger in non-seasonal months if no hunger in seasonal months (indicator)	0.081	0.064
	1667	2890

*Note*: *Hunger in seasonal months (indicator)* is equal to one if the household reported any hunger in January, February, March, or April. *Hunger in seasonal months (count)* is the total number of months the household reported any hunger during this period. *Hunger in seasonal months if no hunger in non-seasonal months (indicator)* is equal to one if the household reported any hunger in January, February, March, or April conditional on the household reporting no hunger in any of the other months. *Hunger in non-seasonal months* variables are defined similarly, but replacing January, February, March, or April with May through December.

Panel B presents statistics for hunger in the other eight months of the year (May to December). Approximately 18 per cent of urban households and 29 per cent of rural households reported at least one month of hunger in the eight months following harvest. This is about half of the number reporting any seasonal hunger, suggesting seasonal hunger is approximately twice as common as non-seasonal hunger. In addition to reporting hunger in the eight months post-harvest less often than in the four months pre-harvest, households that are hungry in the eight months following harvest are hungry for about half as many months on average, even though the period is twice as long. Finally, the last row shows that it is relatively uncommon for households to report hunger in the eight months following harvest but no seasonal hunger. Among urban households that did not report any seasonal hunger, only 8.1 per cent reported any hunger in the other eight months. The number for rural households is just 6.4 per cent.

We next estimate a series of ordered logit regressions – using regressions solely in a descriptive sense – to explore the correlates of seasonal hunger within the sample of agricultural households. The outcome variable for these regressions is a count from 0–4 of the numbers of months in which the household reported any hunger during the three months prior to harvest, plus the month of first harvest by that household. Table 3 presents separate results from ordered logit regressions – presented as odds ratios – for households in survey Waves 1 and 2.

**Table 3.** Seasonal hunger and household and farm characteristics

	Wave 1,Model 1	Wave 1,Model 2	Wave 2,Model 1	Wave 2,Model 2
Age of household head	0.9934*	0.9941	0.9863***	0.9891***
	(0.0038)	(0.0041)	(0.0031)	(0.0035)
Education of household head (years)	0.9238***	0.9177***	0.8881***	0.9054***
	(0.0148)	(0.0157)	(0.0124)	(0.0141)
Male household head	0.7542**	0.8574	0.6235***	0.6027***
	(0.0995)	(0.1180)	(0.0707)	(0.0767)
Household size	1.0971***	1.1043***	1.1124***	1.1040***
	(0.0263)	(0.0288)	(0.0229)	(0.0249)
HH distance to nearest road (km)	1.0156***	1.0126**	1.0146***	1.0106**
	(0.0059)	(0.0063)	(0.0045)	(0.0050)
Total rainfall in reference growing season (mm)	0.9984***	0.9984***	0.9978***	0.9987**
	(0.0004)	(0.0005)	(0.0006)	(0.0006)
Acres owned	0.8177***	0.8228***	1.0000	0.9996
	(0.0315)	(0.0361)	(0.0003)	(0.0002)
Used any organic fertiliser	1.1320	1.0387	1.3085**	1.2516**
	(0.1491)	(0.1473)	(0.1382)	(0.1426)
Used any inorganic fertiliser	0.9818	1.0304	0.7617***	0.6100***
	(0.1456)	(0.1755)	(0.0789)	(0.0691)
Simpson index in previous season (planted acres)		1.6491**		1.6083***
		(0.3253)		(0.2737)
Grew off-season or permanent crop		1.3355**		1.2133*
		(0.1669)		(0.1316)
Poultry (count)		0.9926		0.9994
		(0.0085)		(0.0026)
Other livestock (count)		0.9580**		0.9152***
		(0.0179)		(0.0151)
Stored annual or permanent crop		0.7505*		0.6575***
		(0.1200)		(0.0754)
Remittances and gifts (Kwacha – log+1)		1.0536***		0.9967
		(0.0153)		(0.0112)
Household member worked for wage		1.4992***		1.7105***
		(0.1743)		(0.1773)
Sold annual or permanent crop		0.4625***		1.1102
		(0.1141)		(0.1865)
N	l2052	1804	2589	2199

*Notes*: The outcome variable is a count from 0–4 of the numbers of months in which the household reported any hunger during the three months prior to harvest, plus the month of first harvest by that household. Coefficients are odds ratios from ordered logit regressions.

Standard errors in parentheses. * p < 0.10 ** p < 0.05 *** p < 0.01

Many variables have a significant relationship with the number of months of seasonal hunger a household experiences. The household head being older or male is associated with less seasonal hunger in wave two but only in the first model in wave one. More educated household heads, on the other hand, are strongly associated with less seasonal hunger in both waves. On the other hand, larger households and those farther from the nearest road were associated with more months of seasonal hunger. Acres owned was associated with less seasonal hunger in Wave 1 but not in Wave 2, while both types of fertiliser use are significant in Wave 2 but not Wave 1, with households using inorganic fertiliser on at least one plot experiencing less seasonal hunger, potentially reflecting higher yields. As expected, total rainfall is negatively associated with seasonal hunger.

Household strategies which may help prevent or limit seasonal hunger, such as sending members to look for off-farm employment, may also be responses to insufficient food – a cause and effect our data do not allow us to clearly distinguish. Sale of crops is strongly associated with less seasonal hunger in Wave 1, but not in Wave 2. Ownership of non-poultry livestock and storage of any crop are also associated with less seasonal hunger and these correlations are strongly significant in both waves. Households with greater levels of crop diversity experience more seasonal hunger and cultivation of off-season crops is also positively associated with more months of seasonal hunger, suggesting that crop and season diversity may represent a coping mechanism for households that are seasonally hungry. Similarly, having a household member engaged in wage labour is positively associated with seasonal hunger, perhaps indicating that farm households are more likely to rely on wage labour if they are food-insecure or poor.

Table 4 presents results from the same models but pooling the sample across waves and including enumeration area (EA) and wave fixed effects to control for time-invariant EA-level characteristics which may influence vulnerability to seasonal hunger. Results in Table 4 are similar to those in the initial models without EA and wave fixed effects, but fewer farm characteristics are significant, likely because many of these characteristics are highly spatially correlated. Rainfall is no longer significant and distance to road is only marginally significant in Model 2, but increasing age and education, as well as male gender of the household head remain significantly associated with less seasonal hunger. Crop diversity as measured by the Simpson index is no longer significantly correlated with seasonal hunger, but inorganic fertiliser use is still significant in Model 2. Non-poultry livestock ownership remains significantly correlated with reduced seasonal hunger. We also find a significant relationship between more seasonal hunger and growing off-season or permanent crops, having a household member working for wages, and receipt of remittances, all of which could be coping mechanisms used by households facing bouts of seasonal hunger. Finally, the storage of crops continues to be strongly correlated with less seasonal hunger.

**Table 4.** Seasonal hunger and household and farm characteristics, EA/wave fixed effects

	EA/Wave fixed effects,Model 1	EA/Wave fixed effects,Model 2
Age of household head	0.9880***	0.9913***
	(0.0026)	(0.0030)
Education of household head (years)	0.9223***	0.9303***
	(0.0120)	(0.0127)
Male household head	0.6241***	0.6791***
	(0.0599)	(0.0724)
Household size	1.1132***	1.1071***
	(0.0202)	(0.0219)
HH distance to nearest road (km)	1.0167	1.0225*
	(0.0122)	(0.0129)
Total rainfall in reference growing season (mm)	0.9998	0.9997
	(0.0016)	(0.0020)
Acres owned	0.9993	0.9990
	(0.0006)	(0.0007)
Used any organic fertiliser	1.1215	1.1164
	(0.1090)	(0.1158)
Used any inorganic fertiliser	0.8599	0.8011*
	(0.0886)	(0.0933)
Simpson index in previous season (planted acres)		1.1843
		(0.1930)
Grew off-season or permanent crop		1.2248*
		(0.1296)
Poultry (count)		0.9949
		(0.0048)
Other livestock (count)		0.9168***
		(0.0145)
Stored annual or permanent crop		0.6951***
		(0.0762)
Remittances and gifts (Kwacha – log+1)		1.0286***
		(0.0111)
Household member worked for wage		1.7620***
		(0.1606)
Sold annual or permanent crop		0.8516
		(0.1315)
N	4641	4003

*Notes*: The outcome variable is a count from 0–4 of the numbers of months in which the household reported any hunger during the three months prior to harvest, plus the month of first harvest by that household. Coefficients are odds ratios from ordered logit regressions. Regressions include enumeration area/Wave fixed effects. * p < 0.10 ** p < 0.05 *** p < 0.01

Table 5 presents results from household fixed effects models, which attempt to identify causal relationships between household and farm management characteristics, seasonal hunger, and the date of first crop harvest, testing the hypothesis that seasonally hungry households may cope by harvesting crops early. In the first column, we explore the effects of seasonal hunger on the month of first harvest of any crop, while in the second column we explore these effects on the month of first harvest for maize which is cultivated by 97.4 per cent of agricultural households in Wave 1 and 95.1 per cent in Wave 2, as the first harvest of any crop may include off-season crops where harvest timing is less related to crop maturity. The estimates in Table 5 are from OLS regressions due to the incidental parameters problem. Since the outcome variable is ordinal, however, we also present results from ordered logit regressions in Appendix A (Table A1); conclusions for the coefficients of interest are unchanged.

**Table 5.** Effect of seasonal hunger on household month of first harvest

	(1)	(2)
	Month of FirstHarvest – Any Crop	Month of FirstHarvest – Maize
Seasonal hunger (count)	−0.244***	−0.076***
	(0.042)	(0.024)
Household size	−0.046	−0.028
	(0.039)	(0.023)
Male household head	0.124	0.054
	(0.160)	(0.108)
Acres owned	−0.001	−0.000
	(0.001)	(0.001)
Other livestock (count)	−0.028	−0.003
	(0.020)	(0.009)
Poultry (count)	−0.007	−0.001
	(0.007)	(0.003)
Total rainfall in reference growing season (mm)	−0.001	−0.000
	(0.001)	(0.000)
N	4353	4226
R^2^	0.655	0.782

*Notes*: Standard errors (in parentheses) are clustered at the household level. Regressions include household fixed effects. Both regressions are estimated using OLS. * p < 0.1, ** p < 0.05, *** p < 0.01

In both cases, seasonal hunger in the months before harvest is strongly correlated with the month of first harvest in the current agricultural season. Given the household fixed effects, we cautiously interpret these effects as causal. Each additional month of seasonal hunger is associated with households harvesting their first crop almost a quarter of a month earlier. This suggests that a household experiencing four months of hunger in a row preceding and up to their first harvest may harvest up to a month earlier than a household experiencing zero months of seasonal hunger. Seasonal hunger is also strongly associated with month of first maize harvest, although the magnitude of the effect is only a third of the magnitude of the coefficient in the first model. No other factors are significantly associated with month of first harvest in either model.^10^

## Discussion

Hunger and food insecurity have long been studied by development practitioners and researchers, but much of the focus has been on chronic hunger rather than seasonal hunger. This paper contributes to the growing literature on seasonal hunger in several ways. First, we document the extent of seasonal hunger in Malawi using two waves of a large nationally-representative survey. A large proportion of both urban and rural households experience hunger only in the months before harvests begin. Thus, households affected by seasonal hunger are not just those that suffer from hunger throughout the year, suggesting that different policies may be required for addressing vulnerability to seasonal and chronic hunger.

The first distinction rests with policies that focus on increasing productivity relative to those that focus on smoothing consumption. Our data do not address chronic hunger, but it is reasonable to assume that higher yields, if they reflect true productivity gains, can reduce all types of hunger. The negative association between rainfall in the reference growing season and inorganic fertiliser use and months of seasonal hunger suggests that these factors may support reduced hunger through increased yields. But a household’s ability to smooth income and consumption throughout the year is requisite to fully capturing the value of increased productivity. Because good and poor harvests tend to be spatially clustered (for example, from covariant production shocks such as pests and drought) and the optimal timing of harvest is similar across proximate plots, farmers without a means of storing (including processing) their crop surplus may be forced to sell when prices are low and, as can happen, buy back when prices are high (Amani, 2014; Barrett, 1996; Stephens & Barrett, 2011; Tefera et al., 2011)

In addition to post-harvest storage, descriptive evidence of the correlates of seasonal hunger suggests that households that owned livestock may have been better able to smooth consumption and experience less seasonal hunger. Similarly, age, education, and male gender of the household head are all associated with less seasonal hunger, suggesting that interventions targeting households with younger, less-educated, and female heads may have a greater impact. This result supports Ellis and Maliro’s (2013) finding that social cash transfers targeting poor and vulnerable households in Malawi are an important complement to the FISP, which as currently implemented may not always reach poorer households (Chibwana et al., 2010).

The second distinction is the potential for seasonal hunger to become chronic hunger. In addition to the immediate compromised wellbeing that results from experiencing hunger, short-term coping mechanisms can reasonably be imagined to have negative longer-term health, labour productivity and financial consequences that compound and perpetuate seasonal hunger. Many authors find that seasonal hunger is associated with limiting caloric intake and worsening nutritional outcomes (Christian & Dillon, 2016; Devereux, 2009; Devereux et al., 2008; Hillbruner & Egan, 2008; Longhurst & Payne, 1979; Messer, 1989; Milgroom & Giller, 2013; Sassi, 2015; Vaitla et al., 2009) – with impacts on health and labour productivity (Chambers et al., 1981; Hadley & Patil, 2008). There is evidence of households coping with hunger by eroding assets and incurring debt, increasing financial vulnerability (Ellis & Manda, 2012; Harrigan, 2008; Maxwell, 1996). Our results indicate that agricultural households in Malawi may cope with seasonal hunger by harvesting their crops earlier, with potentially long-term consequences for household members from reduced yields and nutritional value of crops harvested before they are mature.

Our findings suggest that strategies to increase yields (such as the FISP in Malawi) may be ineffective at reducing seasonal hunger and its adverse effects on health and productivity in the absence of policies supporting households to smooth consumption in the lean season. Further, the broader evidence suggests that in the absence of these mechanisms to reduce seasonal hunger, the short-run coping strategies of households may contribute to and extend the next cycle of hunger.

## Notes

1. While definitions differ, in this paper we refer to hunger as insufficient caloric intake (following the United States Department of Agriculture [USDA], 2015).

2. The median number of acres owned is identical in both survey waves, suggesting that the increase in acres owned is driven primarily by increased land holding for households in the top half of the distribution.

3. The qualitative results are unchanged using the current four month seasonal hunger period, the three months prior to but not including the month of first harvest by the household, or the three months prior to and including the month of first harvest.

4. Only agricultural households are included in these regressions, as the models include various farm characteristics.

5. We test for heteroskedasticity in our EA models using the White test. The test strongly rejects the null hypothesis of homoskedasticity. As such, we cluster our standard errors at the EA level, which results in standard errors that are robust to both general heteroskedasticity as well as autocorrelation (at the EA level) (Bertrand, Duflo, & Mullainathan, 2004). To test for multicollinearity, we first constructed a correlation matrix of the main independent variables. The highest pairwise correlation coefficient between independent variables is just 0.3816. In addition, we construct variance inflation factors (VIF) following estimation; the highest VIF is 1.27, which is well below the commonly used cut-off of 10 (Wooldridge, 2009).

6. This diversity index is calculated as: $Simpson=1-\sum \frac{a_{i}}{A}$, where $a_{i}$ is the area devoted to crop *i* and *A* is the total area devoted to crops in the household. Constructed in this way, the variable equals zero when there is no diversity (that is, when there is only one crop planted). An increasing index thus represents increasing diversity (Meng, Smale, Ruffa, Brennan, & Godden, 1999). We do not include area planted to cash crops including tobacco, cotton, and paprika in these calculations.

7. In other words, households in Wave 1 of the survey were asked about the 2008–2009 cropping season, while households in Wave 2 were asked about the 2011–2012 cropping season.

8. Similarly, households in Wave 1 were asked about the 2009–2010 cropping season, while households in Wave 2 were asked about the 2012–2013 cropping season.

9. In the household fixed effects models, we cluster at the household level, which again allows for arbitrary correlation over time and constructs estimates for the standard errors that are valid in the presence of heteroskedasticity, autocorrelation, or both (Bertrand et al., 2004). We also compute the correlation of differences at the household level. In other words, for each independent variable we use in the household fixed effects estimates, we construct the difference in that variable (value in Wave 2 minus value in Wave 1) at the household level. The highest pairwise correlation is only 0.1527. Based on these statistics, we conclude that multicollinearity is not likely to be a problem. Households that split off from Wave 1 panel households and were interviewed only in Wave 2 are not included in these regressions.

10. Household size and count of non-poultry livestock are associated with earlier harvests of any crop in the ordered logit models (Table A1), but not in the OLS models.

## Appendix

**Table A1.** Effect of seasonal hunger on harvest timing, ordered logit

	(1)	(2)
	Month of FirstHarvest – Any Crop	Month of FirstHarvest – Maize
Seasonal hunger (count)	0.538***	0.667***
	(0.039)	(0.059)
Household size	0.883*	0.870
	(0.058)	(0.077)
Male household head	1.411	1.453
	(0.369)	(0.641)
Acres owned	0.998	0.999
	(0.001)	(0.002)
Other livestock (count)	0.934**	0.985
	(0.031)	(0.028)
Poultry (count)	0.980	0.997
	(0.014)	(0.008)
Total rainfall in reference growing season (mm)	0.999	1.000
	(0.001)	(0.001)
N	4353	4226

*Notes*: Standard errors in parentheses. Results are from ordered logit regressions. Coefficients are exponentiated (odds ratios). * p < 0.1, ** p < 0.05, *** p < 0.01
